# Amyotrophic lateral sclerosis established as a multistep process across phenotypes

**DOI:** 10.1111/ene.16532

**Published:** 2024-10-30

**Authors:** Laura Ziser, Ruben P. A. van Eijk, Matthew C. Kiernan, Allan McRae, Robert D. Henderson, David Schultz, Merrilee Needham, Susan Mathers, Pam McCombe, Paul Talman, Steve Vucic

**Affiliations:** ^1^ Institute for Molecular Bioscience, University of Queensland Brisbane Queensland Australia; ^2^ Department of Neurology, UMC Utrecht Brain Centre University Medical Center Utrecht Utrecht The Netherlands; ^3^ Biostatistics and Research Support, Julius Centre for Health Sciences and Primary Care University Medical Center Utrecht Utrecht The Netherlands; ^4^ Neuroscience Research Sydney New South Wales Australia; ^5^ Department of Neurology Royal Brisbane and Women's Hospital Brisbane Queensland Australia; ^6^ Department of Neurology Flinders University of South Australia, and Flinders Medical Centre Bedford Park South Australia Australia; ^7^ Department of Neurology Fiona Stanley Hospital Murdoch Western Australia Australia; ^8^ Centre for Molecular, Medicine Murdoch University Perth Western Australia Australia; ^9^ Department of Neurology Perron Institute for Neurological and Translational Science Nedlands Western Australia Australia; ^10^ Department of Neurology University of Notre Dame Fremantle Western Australia Australia; ^11^ Department of Neurology Calvary Health Care Bethlehem Melbourne Victoria Australia; ^12^ School of Clinical Sciences, Monash University Melbourne Victoria Australia; ^13^ Deakin University, University Hospital Geelong Geelong Victoria Australia; ^14^ Brain and Nerve Research Centre The University of Sydney, Concord Hospital Sydney New South Wales Australia

**Keywords:** amyotrophic lateral sclerosis, incidence, multistep process

## Abstract

**Background and purpose:**

Given the accepted multistep process of disease causation in amyotrophic lateral sclerosis (ALS), the present study was undertaken to determine the number of steps required for disease onset across each of the ALS phenotypes.

**Methods:**

Clinical and demographic data were prospectively accumulated using the Australian Motor Neurone Disease Registry (2005–2016), and age‐specific incidence rates were calculated. Poisson regression was utilized to assess the relationship between log age‐specific incidence and log age of onset, with McFadden's *R* ^2^ used to assess the goodness of fit of the model.

**Results:**

In total, 2647 ALS patients were included, with mean disease‐onset age being 62.2 ± 12.1 years. A linear relationship between log incidence and log age was established across ALS phenotypes, with variable slope estimates: bulbar 5.1 (95% confidence interval [CI] 4.6–5.6); cervical 2.7 (95% CI 2.3–3.0); lumbar 3.5 (95% CI 3.2–3.9); flail arm 4.7 (95% CI 3.9–5.5); flail leg 3.6 (95% CI 2.6–4.5); primary lateral sclerosis 2.7 (95% CI 1.8–3.7). Slope estimates were significantly higher in the bulbar compared to the cervical, lumbar and primary lateral sclerosis phenotypes. McFadden's *R* ^2^ values were >0.4 for all phenotypes indicating excellent model fit.

**Discussion:**

A multistep process has been established across all ALS phenotypes with variable slope estimates, suggesting that the number of steps to develop disease is different across clinical presentations. Identification of mechanisms underlying slope estimate variability could exert pathophysiological significance.

## INTRODUCTION

The pathophysiological mechanisms underlying the development of amyotrophic lateral sclerosis (ALS) appear to be complex, mediated by a complex interaction between genetic, epigenetic and environmental (exposome) factors, resulting in dysfunction of critical molecular pathways and neurodegeneration [1, 2, 3, 4, 5]. Adapted from the Armitage–Doll cancer studies [6], a mathematical modelling approach first demonstrated a linear relationship between the natural log of incidence (as defined by age‐specified rates) and log age of disease onset, indicative of a multistep process in ALS [7]. In a large European cohort, it was reported that sporadic ALS was a six‐step process. Subsequently, a multistep process was reported in large Australian and Asian ALS cohorts, indicating that five to six steps were also required for ALS development [8, 9]. In genetic forms of ALS, a reduced number of steps was reported in European familial ALS cohorts, with two steps associated with the superoxide dismutase 1 (SOD‐1) mutations, three for *C9orf72* hexanucleotide expansion and four for *TARDBP* mutations [10]. These findings suggest that whilst genetic factors predispose to ALS development, epigenetic and environmental factors are also important.

Whilst ALS is characterized by the presence of upper and lower motor neurone (LMN) signs, along with disease progression [11], phenotypic heterogeneity is a well‐recognized feature of ALS [3, 12]. At a clinical level, phenotypic heterogeneity may manifest as variability in disease onset site, with cervical (upper limb), lumbar (lower limb), bulbar (speech and swallow) and thoracic (respiratory) onset regions, as well as difference in rates of disease progression [13]. Additionally, the degree of upper and LMN dysfunction may vary, with LMN (progressive muscular atrophy) predominant and upper motor neurone predominant (primary lateral sclerosis, PLS) modes of presentation reported [3, 5, 12]. For progressive muscular atrophy, flail arm (proximal greater than distal upper limb LMN dysfunction) and flail leg (lower limb predominant with slow evolution) patterns of presentation are well established [14].

Understanding the pathophysiological processes that underlie phenotypic heterogeneity in ALS remains of therapeutic importance. Specifically, establishing whether a multistep process is evident across the different phenotypes, and determining the number of required steps for each phenotype, could potentially identify the source of heterogeneity, thereby enhancing the understanding of ALS pathogenesis. Consequently, the present study employed the previously used Armitage–Doll mathematical modelling approach across different ALS phenotypes. The specific aims were to determine (i) whether a multistep process was evident across the different ALS phenotypes and (ii) whether the number of steps required for development of ALS was different across the phenotypic cohorts, to thereby provide further information about ALS causation.

## METHODS

Data were prospectively accumulated using the Australian Motor Neurone Disease Registry (AMNDR), a national database to which 10 major clinical sites contribute data [12]. Clinical and demographic data, including age of onset and phenotype data, were provided from 2005 to 2016. Phenotypes were defined as follows: (i) ALS cervical (upper limb onset); (ii) ALS lumbar (lower limb onset); (iii) ALS bulbar (bulbar onset); (iv) flail arm; (v) flail leg; and (vi) primary lateral sclerosis (PLS), in accordance with previously published definitions [12, 15].

The Armitage and Doll methodology [6], recently adapted for ALS [7, 8, 9, 10], was utilized to determine whether the specific ALS phenotypes exhibited were a multistep process. Briefly, ALS incidence (*i*) was assumed to be proportional to the risk of undergoing a specific step. In a multistep process, the probability of exposure to step one at *t* years is calculated as *u*1*t*, the second step *u*2*t* and so on until the final disease‐causing step is attained (the *n*th step) with a risk of *u*. Each risk (*u*) is small such that it is not disease causative in isolation. Consequently, the relationship between incidence and risk can be expressed in the following formula [7]:

$$i=u_{1}\times u_{2}\times \ldots \times u_{n-1}\times u_{n}\times t^{n-1}$$

The natural log of incidence then provides the relationship from which the steps can be calculated as follows:

$$\log\left(i\right)=\left(n-1\right)\;\log\left(t\right)+c$$

The constant *c* represents log(*u*_1_ *u*_2_ … *u*_*n*–1_ *u*_*n*_).

Plotting the log of incidence versus the log of age of onset would result in an approximately linear relationship if ALS were a multistep process. The gradient of the linear relationship is *n –* 1, where *n* represents the number of steps required to express the phenotype.

### Statistical analysis

Crude and age‐standardized incidence rates per 100,000 person‐years were calculated in the total ALS cohort and each ALS phenotype. ALS populations 30 years and older were used for calculation of crude and age‐standardized incidence rates. Subsequently, age‐specific incidence rates were calculated for the total ALS cohort and each of the phenotypes. The 30‐ to 75‐year‐old age groups at symptom onset were utilized for calculation of age‐specific incidence rates and were divided into 5‐year epochs. Age groups <30 and >75 years were excluded as per previous studies due to the potential issue of measurement errors or case ascertainment challenges [7]. The midpoint of each age group epoch was used as the age of disease onset and the total number of ALS cases per age epoch was tabulated. Australian population data were provided by the Australian Institute of Health and Welfare (from 2007 to 2016, International Statistical Classification of Diseases and Related Health Problems 10th edition 12.20 and the Australian Standard Population, 2001) and were used to calculate different incidence rates. Age‐specific incidence rates and age of onset were subsequently expressed as the natural log from which the gradient was calculated for the entire cohort as well as each phenotype.

Poisson regression was applied to the standardized incidence data to model the relationship between log incidence and log age of onset for the total ALS cohort. An offset term was included in the model to adjust for the size of the at‐risk population in each age group. The fitted model expresses log incidence as a linear function of log age of onset, providing a slope estimate which can be used to infer the number of steps to develop disease. McFadden's *R* ^2^ value was used to measure the goodness of fit of the model to the standardized incidence data, with values greater than 0.4 interpreted as an excellent fit [16].

Poisson regression was repeated to determine the slope estimate relating log incidence and log age of onset for each phenotype. Analysis of variance was used to assess pairwise differences in slope estimates across the phenotypes with Tukey's honestly significant difference used as a post hoc test. The predicted rate of disease progression was calculated according to a previously reported formula [13].

For the total ALS cohort and each of the phenotypes, the crude incidence rate was also calculated. All data are expressed as mean ± standard deviation; 95% confidence intervals (CIs) were calculated for each variable, the crude incidence rate and slope estimates.

### Role of funding sources

The study was funded by a National Health and Medical Research Council Partnership grant. The funding body was involved in study design, data analysis and interpretation of data, as well as writing of the report. These activities were undertaken by the authors of the paper.

## RESULTS

In total, data were provided on 2659 cases from the AMNDR database, of which 22 (0.8%) were below age 30, 2260 (85%) were between ages 30 and 75 years and 377 (14.2%) were above age 75 years. The mean age of disease onset was 62.2 ± 12.1 years, with mean disease duration at time of registration in the AMNDR database being 24.8 ± 40 months and mean ALS Functional Rating Scale Revised (ALSFRS‐R) 39.0 ± 7.2. The predicted mean rate of disease progression at initial visit was 0.73 ± 0.92. Males represented 60% of the cohort, with cervical phenotype evident in 24.2%, lumbar in 31.4%, bulbar in 27.6%, flail arm in 9.1%, flail leg in 4.1% and PLS in 3.5% of the patients. Bulbar and flail arm phenotypes exhibited the oldest age of onset, whilst the cervical and PLS phenotypes showed the youngest age of onset at the time of registration into the AMNDR database (Table 1). As expected, the disease duration was shortest for the bulbar phenotype and longest for PLS at the time of registration in the AMNDR database (Table 1). The ALSFRS‐R was smallest in the PLS phenotype but was comparable across the remaining phenotypes (Table 1).

**TABLE 1:** Demographic and clinical data for the total amyotrophic lateral sclerosis (ALS) cohort and phenotypes.

	Total ALS cohort	Cervical phenotype	Lumbar phenotype	Bulbar phenotype	Flail arm phenotype	Flail leg phenotype	PLS phenotype
Number (%)	2659	644 (24.2)	835 (31.4)	735 (27.6)	242 (9.1)	109 (4.1)	94 (3.5)
Mean age (years)	62.2 ± 12.1	58.9 ± 13.3	61.5 ± 11.7	65.4 ± 11.1	64.4 ± 11.5	61.1 ± 11.5	59.9 ± 11.5
Males^c^ (%) Females (%)	1586 (60) 1060 (40)	437 (68.3) 203 (31.7)	507 (60.9) 325 (39.1)	348 (47.6) 383 (52.4)	182 (75.8) 58 (24.2)	68 (62.4) 41 (37.6)	44 (46.8) 50 (53.2)
Disease duration (months)	24.8 ± 40	23.6 ± 34	23 ± 47.8	17.1 ± 12.6	31.6 ± 36.2	42.6 ± 61.6	70.8 ± 71.3
ALSFRS‐R	39 ± 7.2	39.0 ± 7.6	39.1 ± 7.2	39.6 ± 6.6	38.2 ± 7.1	40.2 ± 6.7	35.2 ± 7.7
Crude incidence rates/100,000 person‐years^b^ (95% CI)	1.64 (1.43–1.86)	0.39 (0.36–0.43)	0.52 (0.48–0.55)	0.46 (0.42–0.49)	0.15 (0.13–0.17)	0.07 (0.05–0.08)	0.06 (0.05–0.07)
Age‐standardized incidence rates per 100,000 person‐years^b^ (95% CI)	1.55 (1.49–1.62)	0.38 (0.35–0.41)	0.49 (0.45–0.52)	0.43 (0.39–0.46)	0.14 (0.12–0.16)	0.06 (0.04–0.08)	0.06 (0.04–0.07)
Slope estimates^a^ (95% CI)	3.7 (3.5–3.9)	2.7 (2.3–3.0)	3.5 (3.2–3.9)	5.1 (4.6–5.6)	4.7 (3.9–5.5)	3.5 (2.6–4.5)	2.7 (1.8–3.7)
*p* values		<0.001^d^ <0.001^e^	<0.001^d^ 0.01^e^	– 0.46^e^	0.46^d^ –	0.01^d^ 0.07^e^	0.005^d^ 0.04^e^

*Note*: All data are expressed as mean ± standard deviation.

Abbreviations: ALSFRS‐R, Amyotrophic Lateral Sclerosis Functional Rating Scale Revised; CI, confidence interval; PLS, primary lateral sclerosis.

^a^ Slope estimates were calculated for ALS population aged 30–75 years.

^b^ The crude incidence and age‐standardized incidence rates were calculated for the population from 30 to 85 years.

^c^ Gender was not reported in four cervical, three lumbar, four bulbar, two flail arm ALS patients.

^d^ *p* values compared to bulbar phenotype.

^e^ *p* values compared to flail arm phenotype.

The crude incidence rate for the total ALS cohort was 1.64/100,000 person‐years (95% CI 1.43–1.86), whilst the age‐specific incidence rate was 1.55/100,000 person‐years (95% CI 1.49–1.62). Subgroup analysis disclosed that the crude and age‐specific incidence rates were highest for the lumbar phenotype, followed by the bulbar and cervical phenotypes (Table 1). As expected, the atypical phenotypes were less frequently observed, with the flail arm variant form of ALS being the most frequently reported atypical phenotype (Table 1). The crude incidence rates peaked at 70–75 years for the total ALS cohort as well as the cervical, bulbar, flail arm, flail leg and PLS phenotypes. The peak crude incidence rates for the lumbar ALS phenotype were between 65 and 70 years.

To determine whether a multistep process was evident in the ALS phenotypes, the natural log age of onset was regressed with natural log incidence for the ALS population with ages varying from 30 to 75 years in 5‐year epochs. As previously discussed, extreme ages were excluded since estimation of ALS incidence may be imprecise because of small numbers in younger age groups (<30 years) and case under‐ascertainment or generation differences in older age groups (>75 years) [7].

**FIGURE 1:**
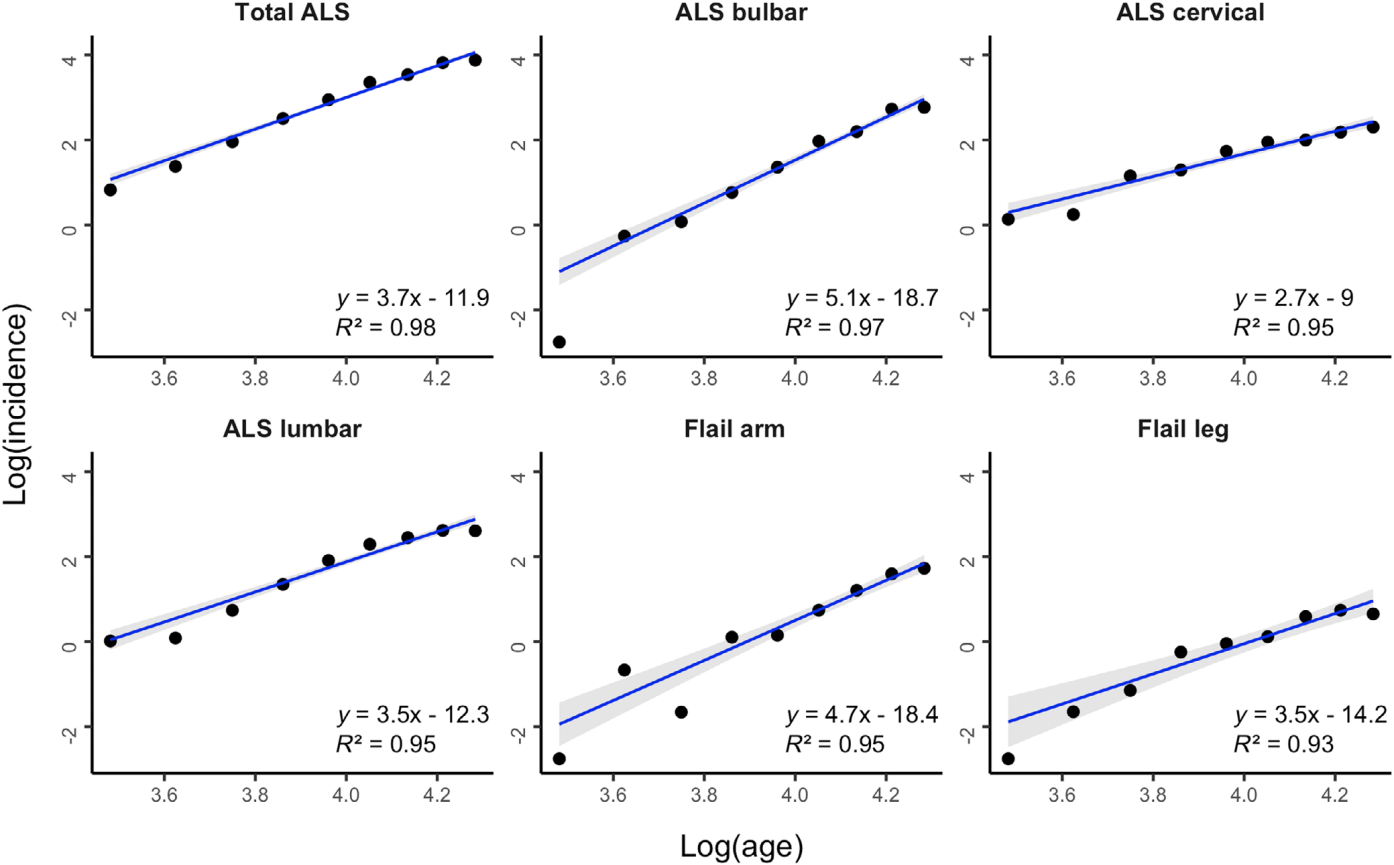
The slope estimates were calculated by expressing the log incidence versus log age of disease onset for the total amyotrophic lateral sclerosis (ALS) cohort (*N* = 2659), as well as the bulbar, cervical (upper limb onset) phenotype, lumbar (lower limb onset), flail arm and flail leg phenotypes. The strong linear relationship suggests a multistep model in all phenotypes. The 95% confidence intervals are highlighted by the grey shading. McFadden's *R* ^2^ value was used to measure goodness of fit of the model to standardized incidence data, with values >0.4 indicating an excellent fit.

**FIGURE 2:**
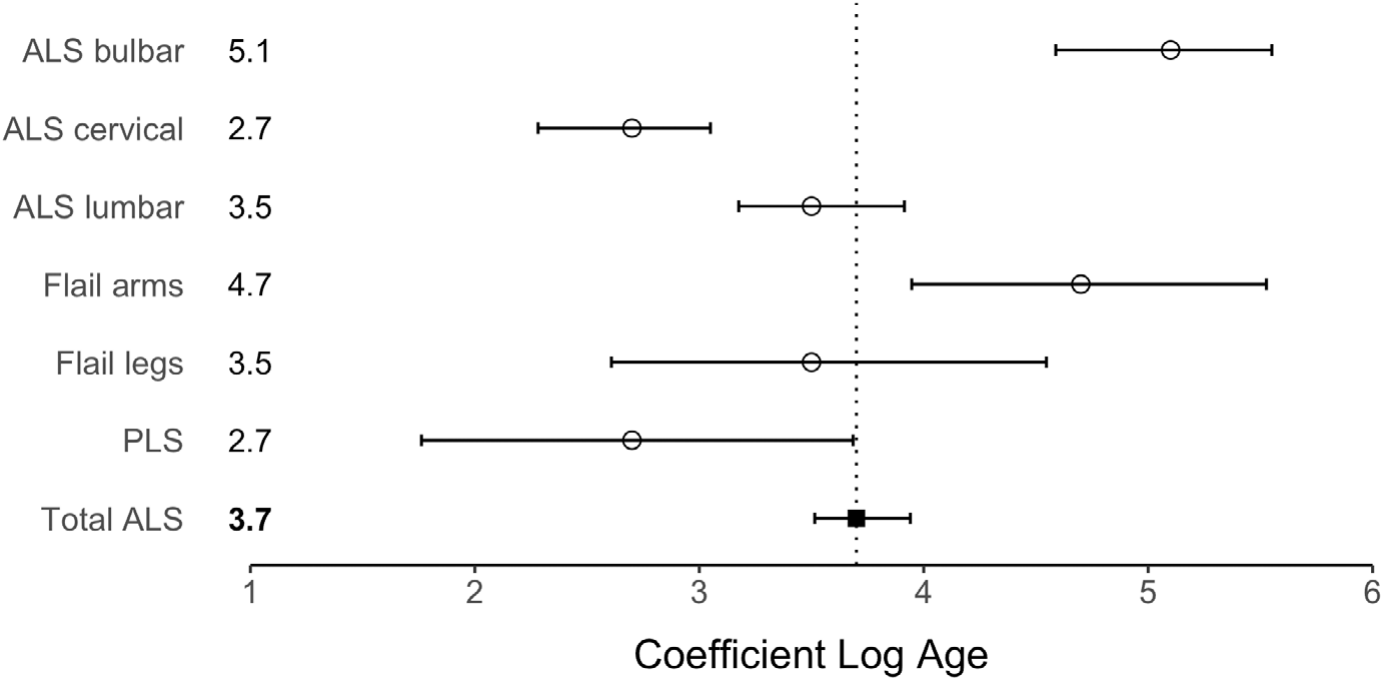
Slope estimates depicted for the total amyotrophic lateral sclerosis (ALS) cohort (*N* = 2659) and the cervical, lumbar, bulbar, flail arm, flail leg and primary lateral sclerosis (PLS) phenotypes. The bars represent 95% confidence intervals.

The slope estimate (*n –* 1) for the total ALS cohort (30–75 years) was 3.7 (95% CI 3.5–3.9), indicating that approximately five steps were required for the development of ALS (Figure 1, Table 1), a finding in keeping with previous studies [8, 9]. Subgroup analysis disclosed a variability of slope estimates across ALS phenotypes (Figure 1). Specifically, the slope estimate for the bulbar phenotype was 5.1 (95% CI 4.6–5.6) and was significantly higher compared to the cervical (2.7, 95% CI 2.3–3.0, *p* < 0.001), lumbar (3.5, 95% CI 3.2–3.9, *p* < 0.001) and PLS phenotypes (2.7, 95% CI 1.8–3.7, *p* = 0.005). The slope estimate was also significantly higher for the flail arm phenotype (4.7, 95% CI 3.9–5.5) compared to cervical (*p* < 0.001), lumbar (*p* = 0.01) and PLS (*p* = 0.04) phenotypes (Figure 2). Additionally, the slope estimate was significantly higher for the lumbar compared to the cervical (*p* = 0.03) phenotype. There were no significant differences in slope estimates comparing other phenotypic combinations.

To assess the extent to which the model that incorporates log age explains log incidence, analogous to *R* ^2^ values used previously for linear regression [7, 8, 9], McFadden's pseudo‐*R* ^2^ values were calculated for the total ALS cohorts and for the ALS phenotypes. The McFadden's pseudo‐*R* ^2^ value for the total ALS cohort was 0.98, indicating that the model regressing log incidence versus log age was an excellent fit. Additionally, McFadden's pseudo‐*R* ^2^ values across the ALS phenotypes indicated an excellent fit of the model regressing log incidence versus log age and were as follows: bulbar (*R* ^2^ = 0.98), cervical (*R* ^2^ = 0.95), lumbar (*R* ^2^ = 0.95), flail arm (*R* ^2^ = 0.95), flail leg (*R* ^2^ = 0.93) and PLS (*R* ^2^ = 0.76) (Figure 2). The findings suggest a strong linear relationship between log incidence and log age, and thereby that ALS is a multistep process irrespective of phenotype.

## DISCUSSION

The present study has established a linear relationship between the log incidence and log age of onset across different ALS phenotypes, suggesting the presence of a multistep process. Perhaps unexpectedly, the slope estimates varied across the phenotypes, being smallest for cervical and PLS and highest for the bulbar phenotype. Specifically, whilst a four‐step process was suggested for the cervical and PLS phenotypes, a six‐step process was evident in the bulbar phenotype. Separately, five steps were required for the lumbar and flail leg, whilst a six‐step process was suggested for the flail arm phenotype. At a pathophysiological level, the present study implies a similarity of mechanisms across phenotypes, namely a multistep process, although the number of steps is varied which could contribute to the phenotypic variability of ALS. The implications of the findings in understanding ALS pathogenesis, and in particular the emergence of heterogeneity, are further discussed.

### Pathophysiological mechanisms underlying ALS

A complex interaction between genetic, epigenetic and environmental (exposome) factors appears to underlie ALS pathogenesis in a multistep process [2, 3, 5, 7, 9, 17]. Whilst subtle differences in slope estimates were reported between male and female ALS patients [9], there was no analysis of whether slope estimates varied between ALS phenotypes. The present study established a multistep process across the recognized ALS phenotypes by demonstrating a strong linear relationship between log incidence and log age of onset. Somewhat surprisingly, the slope estimates were significantly higher for the bulbar phenotype compared to cervical and lumbar phenotypes, despite a more adverse prognosis for the bulbar onset ALS patients [12]. In addition, the PLS phenotype exhibited a relatively small number of steps (*N* = 3) compared to the more aggressive phenotypes. Consequently, the present study suggests that pathogenic mechanisms underlying the development of ALS may be distinct from mechanisms driving disease progression and an adverse prognosis. Moreover, the differences in slope estimates could contribute to the phenotypic variability evident in ALS.

An explanation for slope differences across ALS phenotypes, which are discordant with prognostic expectations, is not immediately apparent. The phenotypic differences in slope estimates may be related to the expression of specific genetic mutations and gene modifiers which are known to influence the ALS phenotype, such as site of disease onset, rate of disease progression, degree of upper or LMN dysfunction as well as the extent of cognitive dysfunction [18, 19, 20]. Of relevance, the expression of genetic mutations at specific stages in life, as well as variability of expression in specific body regions, could also account for the slope estimate differences across the ALS phenotype [21]. Additionally, an interaction between genetic mutations and in utero or neonatal environmental factors could represent initial pathogenic triggers [22, 23], resulting in a vulnerability of specific motor neuronal networks. Subsequently, when exposed to environmental factors, the vulnerable neuronal networks would be more prone to degeneration resulting in specific phenotypes. Underscoring this notion is the impact of specific genetic mutations on the number of steps required for ALS development [10], whereby inheritance of SOD‐1 mutations necessitates two additional steps to express a phenotype, *C9orf72* expansions require three and *TARDBP* mutations a further four steps. Utilizing cell and molecular based techniques across ALS phenotypes could provide further insights into the mechanisms underlying the differences in slope estimates that would be of pathogenic significance.

An important consideration for explaining differences in the slope estimates across ALS phenotypes could be the interaction between genetic and environmental factors [2, 3]. A multistep model posits that multiple ‘hits’ are required to trigger ALS, explaining the importance of environmental factors in ALS aetiology. Prolonged exposure to a variety of environmental factors, including pesticides, occupational exposures, sports and physical activity, metals, electromagnetic fields, air pollution, head trauma, alterations in gut microbiome, as well as diet and lifestyle factors, could all contribute to ALS development [2, 21, 24]. Whilst in isolation these environmental factors are insufficient to trigger ALS [2], sustained exposure and interactions with genetic, molecular, neuronal and non‐neuronal processes in a multistep process may combine to trigger ALS development.

The exposure to specific environmental factors could contribute to phenotypic heterogeneity, although the mechanisms remain to be fully elucidated. Specifically, bulbar onset ALS has been linked to increased radiation exposure [25], specific occupations [26] and sports [24]. Alternatively, exposure to volatile organic compounds, combustion and diesel exhaust as well as electromagnetic radiation has been linked to cervical onset disease [25]. The magnitude and rate of exposure of ALS patients to specific environmental factors, combined with a vulnerability to heritability, could contribute to the ‘step’ differences across different phenotypes. Further studies exploring the genomic and proteomic profiles along with environmental exposure in ALS patients and their parents may uncover the precise sequence of steps involved in ALS disease onset and phenotype variability, thereby resulting in a clearer understanding of pathogenesis.

Significant differences in gender have been reported in ALS phenotypes, with the smallest male:female ratio reported in bulbar onset phenotypes [14]. Differences in slope estimates have been established between female (seven steps) and male (six steps) ALS patients [9]. The gender effects could be related to the liability threshold model of disease, whereby female ALS patients carry a higher disease burden and consequently require more steps to develop the disease [27]. Separately, modifier effects of higher oestrogen levels on vital molecular processes in ALS could also contribute to a higher number of steps in female patients. Consequently, it could be argued that similar mechanisms are operative in bulbar onset as in female ALS patients, thereby resulting in a higher number of steps.

Cortical hyperexcitability has been identified as an important pathophysiological feature of ALS [23, 28, 29, 30], correlating with LMN degeneration and functional decline [31, 32]. It would seem attractive to suggest that cortical hyperexcitability could account for slope differences across ALS phenotypes, especially given that cortical hyperexcitbaility may be more prominent in the upper limb onset [31, 33], flail arm [34] and flail leg [35] ALS phenotypes. A potential argument against this notion pertains to the fact that cortical inexcitability is a feature of PLS, which only requires three steps. Consequently other pathogenic mechanisms may also contribute to slope estimate differences across ALS phenotypes. Uncovering these processes could enhance the understanding of ALS pathogenesis and lead to the development of therapeutic approaches that would ultimately modify disease progression.

A potential limitation of the present study relates to the absence of information regarding genetic status in the included ALS cohort. Documentation of SOD‐1, FUS and *TARDBP* mutations, or co‐inheritance of gene modifiers, was not documented in the AMNDR. Moreover, the registry was established prior to identification of the *C9orf72* hexanucleotide expansion. Whilst a potential contribution of specific genetic mutations to slope estimates across the phenotypes could not be discounted, given that point mutations (SOD‐1, FUS or *TARDBP*) represent ~2% of the Australian ALS cohort and *C9orf72* hexanucleotide expansion ~10% [4], it is unlikely that inadvertent inclusion of the genetic cohorts would have appreciably impacted the present findings. Repeating the study in a separate database with known genetic status would be important to determine whether the association between step numbers and ALS phenotypes holds true. It could also be argued that differences in categorization of ALS phenotypes across multiple centres could have contributed to the current findings. This seems unlikely given that all sites formed the AMNDR database and agreed to predefined input variables, including the ‘ALS phenotype’. Alternatively, a one‐step process occurring in a cluster of cells could also account for the observed relationship between natural log incidence and log age [7]. Consequently, in the bulbar onset phenotype the pathogenic processes occurred in seven cells, whilst in the cervical phenotype these occurred in five cells, and so forth, followed by prion‐like evolution, although the pathogenic processes occurred at the same time.

#### In conclusion

The present study established a multistep process across the ALS phenotypes, with the slope estimates being highest for the bulbar onset phenotype (requiring seven steps) and smallest in the cervical phenotype. Intriguingly, differences in slope estimates were not congruent with the reported phenotypic prognosis [12], suggesting that pathogenic factors the mediate ALS onset may be different from those governing disease progression. The present findings were congruent with previous studies reporting a multistep process in ALS [7, 8, 9]. Identifying the mechanisms that influence the number of pathogenic steps in ALS phenotypes would enhance the understanding of disease heterogeneity and pathogenesis, ultimately leading to novel therapeutic approaches.

## AUTHOR CONTRIBUTIONS

**Laura Ziser:** Conceptualization; methodology; software; data curation; formal analysis; writing – original draft; visualization. **Ruben P. A. van Eijk:** Methodology; data curation; formal analysis; writing – review and editing; visualization; validation. **Matthew C. Kiernan:** Conceptualization; methodology; data curation; writing – review and editing. **Allan McRae:** Writing – review and editing; formal analysis; validation; methodology. **Robert D. Henderson:** Supervision; writing – review and editing; data curation. **David Schultz:** Data curation; supervision; writing – review and editing. **Merrilee Needham:** Supervision; data curation; writing – review and editing. **Susan Mathers:** Writing – review and editing; supervision; data curation. **Pam McCombe:** Writing – review and editing; supervision. **Paul Talman:** Conceptualization; methodology; validation; formal analysis; data curation; supervision; writing – review and editing. **Steve Vucic:** Conceptualization; methodology; software; data curation; investigation; validation; formal analysis; writing – review and editing; supervision; project administration.

## FUNDING INFORMATION

NHMRC Partnership grant.

## CONFLICT OF INTEREST STATEMENT

MCK reports being director of a company that holds equity in Clene Nanomedicine. MCK was Editor‐in‐Chief of the *Journal of Neurology, Neurosurgery and Psychiatry* (BMJ Publishers, UK). He is Director of the Forefront Motor Neurone Disease and Frontotemporal Dementia Clinic and has directed clinical trials on behalf of the University of Sydney as Principal or Chief Investigator, with funds managed through the Central Clinical School. He is President of the Brain Foundation Australia. MN reports receiving consultancy fees CSL, Sanofi‐Aventis, Abauno. RH reports receiving honoraria form CSL. SV reports being director of a company that holds equity in Clene Nanomedicine. SV reports receiving fees for advisory board participation from Biogen. SV is chair of the Brain Foundation Australia scientific committee. The other authors report no conflicts of interest.

## Data Availability

The data that support the findings of this study are available from the corresponding author upon reasonable request.

## References

[1] Geevasinga N , Menon P , Özdinler PH , Kiernan MC , Vucic S . Pathophysiological and diagnostic implications of cortical dysfunction in ALS. Nat Rev Neurol. 2016;12(11):651‐661. 27658852 10.1038/nrneurol.2016.140

[2] Goutman SA , Savelieff MG , Jang DG , Hur J , Feldman EL . The amyotrophic lateral sclerosis exposome: recent advances and future directions. Nat Rev Neurol. 2023;19(10):617‐634. 37709948 10.1038/s41582-023-00867-2 PMC11027963

[3] Feldman EL , Goutman SA , Petri S , et al. Amyotrophic lateral sclerosis. Lancet. 2022;400(10360):1363‐1380. 36116464 10.1016/S0140-6736(22)01272-7 PMC10089700

[4] Kiernan MC , Vucic S , Talbot K , et al. Improving clinical trial outcomes in amyotrophic lateral sclerosis. Nat Rev Neurol. 2020;17:104‐118. 33340024 10.1038/s41582-020-00434-z PMC7747476

[5] Kiernan MC , Vucic S , Cheah BC , et al. Amyotrophic lateral sclerosis. Lancet. 2011;377(9769):942‐955. 21296405 10.1016/S0140-6736(10)61156-7

[6] Armitage P , Doll R . The age distribution of cancer and a multi‐stage theory of carcinogenesis. Br J Cancer. 1954;8(1):1‐12. 13172380 10.1038/bjc.1954.1 PMC2007940

[7] Al‐Chalabi A , Calvo A , Chio A , et al. Analysis of amyotrophic lateral sclerosis as a multistep process: a population‐based modelling study. Lancet Neurol. 2014;13(11):1108‐1113. 25300936 10.1016/S1474-4422(14)70219-4 PMC4197338

[8] Vucic S , Higashihara M , Sobue G , et al. ALS is a multistep process in south Korean, Japanese, and Australian patients. Neurology. 2020;94(15):e1657‐e1663. 32071166 10.1212/WNL.0000000000009015 PMC7251515

[9] Vucic S , Westeneng HJ , Al‐Chalabi A , Van Den Berg LH , Talman P , Kiernan MC . Amyotrophic lateral sclerosis as a multi‐step process: an Australia population study. Amyotroph Lateral Scler Frontotemporal Degener. 2019;20(7–8):532‐537. 31284763 10.1080/21678421.2018.1556697

[10] Chio A , Mazzini L , D'Alfonso S , et al. The multistep hypothesis of ALS revisited: the role of genetic mutations. Neurology. 2018;91(7):e635‐e642. 30045958 10.1212/WNL.0000000000005996 PMC6105040

[11] Shefner JM , Al‐Chalabi A , Baker MR , et al. A proposal for new diagnostic criteria for ALS. Clin Neurophysiol. 2020;131:1975‐1978. 32387049 10.1016/j.clinph.2020.04.005

[12] Talman P , Duong T , Vucic S , et al. Identification and outcomes of clinical phenotypes in amyotrophic lateral sclerosis/motor neuron disease: Australian National Motor Neuron Disease observational cohort. BMJ Open. 2016;6(9):e012054. 10.1136/bmjopen-2016-012054 PMC5051496 27694488

[13] Labra J , Menon P , Byth K , Morrison S , Vucic S . Rate of disease progression: a prognostic biomarker in ALS. J Neurol Neurosurg Psychiatry. 2016;87:628‐632. 26152368 10.1136/jnnp-2015-310998

[14] Wijesekera LC , Mathers S , Talman P , et al. Natural history and clinical features of the flail arm and flail leg ALS variants. Neurology. 2009;72(12):1087‐1094. 19307543 10.1212/01.wnl.0000345041.83406.a2 PMC2821838

[15] Chio A , Canosa A , Gallo S , et al. ALS clinical trials: do enrolled patients accurately represent the ALS population? Neurology. 2011;77(15):1432‐1437. 21956723 10.1212/WNL.0b013e318232ab9b

[16] McFadden D . Conditional logit analysis of qualitative choice behavior. In: Zarembka P , ed. Frontiers in Econometrics. Academic Press; 1974.

[17] Bennett SA , Tanaz R , Cobos SN , Torrente MP . Epigenetics in amyotrophic lateral sclerosis: a role for histone post‐translational modifications in neurodegenerative disease. Transl Res. 2019;20419‐30:19‐30. 10.1016/j.trsl.2018.10.002 PMC6331271 30391475

[18] Moglia C , Calvo A , Canosa A , et al. Cognitive and behavioral features of patients with amyotrophic lateral sclerosis who are carriers of the *TARDBP* pathogenic variant. Neurology. 2024;102(4):e208082. 38261982 10.1212/WNL.0000000000208082 PMC10962913

[19] Martinelli I , Ghezzi A , Zucchi E , et al. Predictors for progression in amyotrophic lateral sclerosis associated to SOD1 mutation: insight from two population‐based registries. J Neurol. 2023;270(12):6081‐6092. 37668704 10.1007/s00415-023-11963-0

[20] Chiò A , Moglia C , Canosa A , et al. Association of copresence of pathogenic variants related to amyotrophic lateral sclerosis and prognosis. Neurology. 2023;101(1):e83‐e93. 37202167 10.1212/WNL.0000000000207367 PMC10351316

[21] Al‐Chalabi A , Hardiman O . The epidemiology of ALS: a conspiracy of genes, environment and time. Nat Rev Neurol. 2013;9(11):617‐628. 24126629 10.1038/nrneurol.2013.203

[22] Kiernan MC , Ziemann U , Eisen A . Amyotrophic lateral sclerosis: origins traced to impaired balance between neural excitation and inhibition in the neonatal period. Muscle Nerve. 2019;60(3):232‐235. 31233613 10.1002/mus.26617

[23] Eisen A , Kiernan M , Mitsumoto H , Swash M . Amyotrophic lateral sclerosis: a long preclinical period? J Neurol Neurosurg Psychiatry. 2014;85(11):1232‐1238. 24648037 10.1136/jnnp-2013-307135

[24] Chio A , Benzi G , Dossena M , Mutani R , Mora G . Severely increased risk of amyotrophic lateral sclerosis among Italian professional football players. Brain. 2005;128(Pt 3):472‐476. 15634730 10.1093/brain/awh373

[25] Goutman SA , Boss J , Godwin C , Mukherjee B , Feldman EL , Batterman SA . Associations of self‐reported occupational exposures and settings to ALS: a case–control study. Int Arch Occup Environ Health. 2022;95(7):1567‐1586. 35593931 10.1007/s00420-022-01874-4 PMC9424174

[26] Farrugia Wismayer M , Borg R , Farrugia Wismayer A , et al. Occupation and amyotrophic lateral sclerosis risk: a case–control study in the isolated island population of Malta. Amyotroph Lateral Scler Frontotemporal Degener. 2021;22(7–8):528‐534. 33821701 10.1080/21678421.2021.1905847

[27] Larson TC , Kaye W , Mehta P , Horton DK . Amyotrophic lateral sclerosis mortality in the United States, 2011–2014. Neuroepidemiology. 2018;51(1–2):96‐103. 29990963 10.1159/000488891 PMC6159829

[28] Menon P , Geevasinga N , Yiannikas C , Howells J , Kiernan M , Vucic S . The sensitivity and specificity of threshold‐tracking transcranial magnetic stimulation for the diagnosis of amyotrophic lateral sclerosis: a prospective study. Lancet Neurol. 2015;14:14478‐14484. 10.1016/S1474-4422(15)00014-9 25843898

[29] Vucic S , Nicholson GA , Kiernan MC . Cortical hyperexcitability may precede the onset of familial amyotrophic lateral sclerosis. Brain. 2008;131(Pt 6):1540‐1550. 18469020 10.1093/brain/awn071

[30] Menon P , Kiernan MC , Vucic S . Cortical hyperexcitability precedes lower motor neuron dysfunction in ALS. Clin Neurophysiol. 2015;126(4):803‐809. 25227219 10.1016/j.clinph.2014.04.023

[31] Vucic S , Kiernan MC . Novel threshold tracking techniques suggest that cortical hyperexcitability is an early feature of motor neuron disease. Brain. 2006;129:2436‐2446. 16835248 10.1093/brain/awl172

[32] Van den Bos MAJ , Higashihara M , Geevasinga N , Menon P , Kiernan MC , Vucic S . Imbalance of cortical facilitatory and inhibitory circuits underlies hyperexcitability in ALS. Neurology. 2018;91(18):e1669‐e1676. 30282772 10.1212/WNL.0000000000006438

[33] Menon P , Yiannikas C , Kiernan MC , Vucic S . Regional motor cortex dysfunction in amyotrophic lateral sclerosis. Ann Clin Transl Neurol. 2019;6(8):1373‐1382. 31402622 10.1002/acn3.50819 PMC6689694

[34] Vucic S , Kiernan MC . Abnormalities in cortical and peripheral excitability in flail arm variant amyotrophic lateral sclerosis. J Neurol Neurosurg Psychiatry. 2007;78:849‐852. 17210625 10.1136/jnnp.2006.105056 PMC2117729

[35] Menon P , Geevasinga N , Yiannikas C , Kiernan MC , Vucic S . Cortical contributions to the flail leg syndrome: pathophysiological insights. Amyotroph Lateral Scler Frontotemporal Degener. 2016;17(5–6):389‐396. 26888565 10.3109/21678421.2016.1145232

